# Misreporting of Energy Intake in the 2007 Australian Children’s Survey: Identification, Characteristics and Impact of Misreporters 

**DOI:** 10.3390/nu3020186

**Published:** 2011-02-08

**Authors:** Anna M. Rangan, Victoria M. Flood, Timothy P. Gill

**Affiliations:** 1 Cluster for Public Health Nutrition, Boden Institute of Obesity, Nutrition and Exercise, The University of Sydney, Level 2, Medical Foundation Building K25, 2006 NSW, Australia; Email: t.gill@sydney.edu.au; 2 School of Health Sciences, University of Wollongong, 2522 NSW, Australia; Email: vflood@uow.edu.au

**Keywords:** children, nutrition survey, energy intake, child nutritional physiological phenomena, Australia

## Abstract

Misreporting of energy intake (EI) is a common problem in national surveys. The aim of this study was to identify misreporters using a variety of criteria, examine the impact of misreporting on the association between EI and weight status, and to define the characteristics of misreporters in the 2007 Australian Children’s Survey. Data from the 2007 Australian Children’s Survey which included 4800 children aged 2–16 years were used to examine the extent of misreporting based on EI, physical activity level (PAL), age, gender, height and weight status. Three options for identifying misreporters using the Goldberg cut-offs were explored as was direct comparison of EI to energy expenditure (TEE) in a subset of children. Linear regression was used to determine the impact of misreporting on the association between EI and weight status. The prevalence of under-reporting among all children varied from 5.0% to 6.7%, and over-reporting from 1.6% to 3.0% depending on the option used. Direct comparison of EI to TEE revealed similar results. Regression analysis showed that excluding misreporters provided the best model to examine cross-sectional associations between EI and BMI. Characteristics associated with under-reporting included older age, female, higher BMI, higher PAL, living in an urban location, lower parental education level and feeling unwell on the survey day. Over-reporting was more common among children with a lower BMI and lower PAL. In conclusion, misreporting of EI is present among various subgroups of the 2007 Australian Children’s Survey. The impact of misreporting on the association between EI and body weight should be recognised by users of this survey.

## 1. Introduction

Misreporting of energy intake (EI) comprising both under- and over-reporting is a common problem in dietary surveys and has been well described in a number of national surveys among adults [1,2,3,4,5,6]. For example in the 1995 National Nutrition Survey, 12% of men and 21% of women were classified as under-reporting their EI [3]. The degree of misreporting varies according to a number of factors including the dietary assessment method used, physical activity level (PAL) used, application of the 95% or 99% confidence limits, and characteristics of the population. Under-reporting can be due to under-eating, when respondents eat less than usual, and under-recording, when respondents fail to record all the foods consumed or underestimate the amounts consumed. Over-reporting of EI is less well studied and tends to be less prevalent than under-reporting.

Biases are introduced into a dietary survey when respondents with certain characteristics (e.g., overweight) under-report their food and EIs relative to their counterparts (e.g., those with a healthy weight). Apart from misreporting EIs, there is also an increased likelihood of misreported nutrient intakes such as fat, sugar, fibre and micronutrients. Associations between food and nutrient intakes and body weight status derived from datasets including misreporters are therefore likely to be flawed [7,8].

The validity of reported EI is often assessed by comparing EI to total energy expenditure (TEE). Assuming that body weight is stable, EI is equal to TEE, hence TEE can be used to assess EI. There are several methods to measure TEE, with the doubly-labelled water (DLW) method being considered the gold standard. However, due to its expense and need for extensive resources this method has not been a routine part of national dietary collection surveys. Other methods used to assess TEE include heart rate monitoring, use of accelerometers, and self-reported physical activity data. The Goldberg approach is a widely used alternative to identify invalid reports of EI and can be used when TEE is not available or is approximated [9]. This approach compares TEE with EI when both are expressed as a multiple of basal metabolic rate (BMR): EI/BMR *=* TEE/BMR (with BMR estimated from equations). The TEE/BMR ratio is also known as PAL. Confidence limits (CL) of agreement or Goldberg cut-offs are applied, based on variation in EI, BMR and PAL, to identify individuals with intakes that are unlikely to represent valid data. PAL can vary significantly between individuals from 1.2 for those chair-bound or bedridden, to 1.55 for those with low activity levels to 2.4 for professional athletes [10]. If a PAL is not available, a minimum plausible value of 1.55 is usually assigned to all individuals, proposed by FAO/WHO/UNU [11] based on the assumption that subjects have a low activity level (*i.e.*, normally active but sedentary). However, assigning a single PAL to a group results in relatively poor sensitivity with only 50% of under-reporters being detected in dietary surveys of adults [12]. Consequently, the use of individual PALs is highly recommended to identify both under- and over-reporting as lower and upper cut-offs can be applied with more confidence. This has been demonstrated to improve sensitivity while specificity remains high [12]. 

There are no clear guidelines how to use and interpret data obtained from misreporters. As the inclusion of misreporters can produce erroneously low results for habitual food or nutrient intakes and therefore confound the relationship between dietary intakes and diet-related diseases, it is important to know more about the nature and extent of misreporting, who it affects and any bias resulting from it. The 2007 Australian National Children’s Nutrition and Physical Activity Survey (2007 Australian Children’s Survey) is the latest representative national dietary survey comprising over 4000 children aged 2–16 years. This survey is well suited to examine the issue of misreporting due to the availability of data on dietary intake, measured heights and weights, and PAL. 

The objectives of this paper were (1) to identify misreporters (under- and over-reporters) using a variety of different methods and cut-offs; (2) to describe the characteristics of misreporters; and (3) to explore the impact of misreporters on the relationship between EI and BMI using data from the 2007 Australian Children’s Survey. 

## 2. Methods

### 2.1. 2007 Australian Children’s Survey

Data for the 2007 Australian Children’s Survey, collected by the Commonwealth Scientific and Industrial Research Organisation (CSIRO) and the University of South Australia, were obtained with permission from the Australian Social Sciences Archives [13]. In brief, households with children aged 2–16 years were randomly selected using a stratified quota sampling scheme by postcodes. Private dwellings from selected postcodes were recruited to the survey using Random Digital Dialling. Only one child per household was selected for the survey. The response rate for this survey was 40% of eligible households [14]. 

Out of two 24-h recalls, only the first recall interview (a computer-assisted personal interview, CAPI) was used for this secondary analysis as this allows comparison to previous national surveys, which have only collected one day recall for most people. A total of 4826 children completed the CAPI. A three-pass 24-h recall method was used to record all food and beverage intakes on the day prior to each interview from midnight to midnight. A food model booklet was provided to estimate food portion sizes. All interviews were conducted by trained interviewers between 22 February 2007 and 30 August 2007 and were intended to represent different days of the week. Dietary data were collected from the primary care-giver for children aged 2–8 years (*n =* 2452) whereas children aged 9 years and older (*n =* 2374) reported their own food intakes [14]. Dietary data were translated into nutrient intake data using a specifically designed nutrient database, AUSNUT 2007 (Australian Food and Nutrient Database) [15]. 

### 2.2. PAL

Physical activity was measured among 9–16 year old children using a validated use-of-time tool; Multimedia Activity Recall for Children and Adolescents (MARCA) [16]. Each child recalled 4 days worth of activities (2 days prior to each of the dietary recalls) in time slices as fine as 5 min. Energy expenditure was calculated based on the reported activities and expressed as PAL*.* The average of 4 days of PAL was calculated per child (*n =* 2242). Children who did not participate in the PAL dairies were assigned the mean PAL of the 9–16 year old group, which was 1.65 (SD 0.25), and referred to as estimated PAL. This applied to 132 children aged 9–16 years and 2452 children aged 2–8 years. 

### 2.3. Under- and Over-Reporters

The extent of misreporting was examined among all children using three different criteria: Option 1, applying a single PAL of 1.55 to all children; Option 2, applying the individual PAL; using the measured PAL (for children 9 years and over) or estimated PAL for children with no measured PAL (median PAL of 1.65); Option 3, applying the individual PAL (as for option 2) but with wider cut-offs for the estimated values where no PAL measurement is available (using lower cut-off PAL of 1.50 and upper cut-off PAL of 1.80 which represent the 25th and 75th percentiles of those children with measured PAL). A fourth option, direct comparison, was applied to only the subset of children who had measured PALs (*n =* 2242).

#### 2.3.1. Goldberg Cut-offs

The Goldberg cut-off values were applied to exclude under-reporters and over-reporters, based on PAL and compared with the ratio of EI to BMR. BMR was calculated using the Schofield equations for children based on age, gender, height and weight [17].

The cut-off values are the CL of agreement between EI:BMR and PAL, and are created based on the coefficients of variation (CV) of subjects’ EI (CV_wEI_), the accuracy of the measurement of their basal metabolic rate (CV_wB_), and the total variation in PAL (CV_tP_). Approximate values for these CV parameters have been estimated by Black based on the pooled mean of several studies and are suitable to substitute into the Goldberg equation [18].

The equation used to calculate the cut-offs is [18]: 


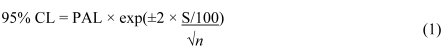


where *n* = number of subjects (*i.e.*, 1 based on individual, not group, requirements)

  exp = exponential funtion 

  S = factor taking into account the variation in EI, BMR and PAL

   = √[CV^2^_wEI_/d + CV^2^_wB_ + CV^2^_tP_]

   = 28.7

   where CV_wEI_ = within-subject variation in EI (23%)

        d = number of days of dietary assessment (*i.e.*, 1)

        CV_wB_ *=* variation in repeat BMR measures (8.5%)

        CV_tP_ = total variation in PAL (15%)

After substituting these values into the equation, the lower and upper 95% CL or cut-offs generated were PAL × 0.562 and PAL × 1.778, respectively. For example, for children with a PAL of 1.65, the 95% CL were 0.93 and 2.93. Diet recalls with EIs below the cut-offs (at 95% CL) were considered under-reporters, recalls with EIs between the cut-offs were plausible reporters and those with EIs above the cut-offs were considered over-reporters. 

#### 2.3.2. Direct Comparison

This method can be applied to children whose PAL was measured using the 4 day physical activity diaries as it compares the ratio of EI to TEE directly. The expected ratio of EI to TEE is 1.00; those reporting less than 1.00 are assumed to have under-reported EI to some degree while those reporting more than 1.00 are assumed to have some degree of over-reporting EI. The 95% CL applied to the ratio of EI to TEE include variation of within-subject EI (CV_wEI_) and variation in TEE (CV_wEE_) and were calculated as [12]:


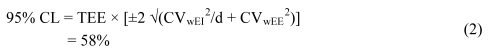


where TEE = BMR × measured PAL

     d = number of days of dietary assessment (*i.e.*, 1)

     CV_wEI_ = within-subject variation in EI (23%)

     CV_wEE_ = within-subject variation in TEE (17.8%; including CV for BMR at 8.5% and CV for PAL at 15.6%, as calculated from the current study) 

Using the direct comparison method, under-reporters were defined as those with EI/TEE ratios less than 0.42, while over-reporters had EI/TEE ratios over 1.58.

### 2.4. Statistical Methods

Descriptive analyses were calculated for the various methods of classifying under- and over-reporting. ANOVA and chi square statistics were performed to study potential differences between under-reporters, plausible reporters and over-reporters in age (years), gender, parental education (highest qualification attained by primary caregiver; school/certificate or diploma/degree), area of residence (urban or rural), day of the week of dietary data collection (weekday or weekend day), BMI (weight/height^2^, continuous variable), PAL (continuous variable) and unusual intake on survey day (due to feeling unwell or no comment). All data were weighted to represent the Australian population in terms of age, gender and region. BMI *z*-scores and BMI-for-age were calculated using the Centers for Disease Control growth charts [19]. 

Linear regression modelling was used to examine the impact of misreporting on the association between reported EI and weight status. The first model included all respondents (including misreporters) and the other models included only plausible respondents (identified from the three options). Reported EI was the independent variable, and age, sex and BMI *z*-score were the dependent variables. Potentially confounding variables such as parental education level, area of residence and PAL were not included in the modelling as these were found to be insignificant. All analyses were conducted using SPSS version 17.0. *p* values < 0.05 were considered statistically significant.

## 3. Results

### 3.1. Identifying Potential Misreporters

The prevalence of misreporting based on the use of different PALs is reported in Table 1. Using cut-offs based on PAL of 1.55 (option 1), under-reporting was found in 5% of children, which was the lowest level among the three options applied. Under-reporting was particularly low for boys and girls aged 2 to 8 years (less than 2%), then rose to 5–6% for children aged 9 to 13 years and 8–15% for children aged 14 to 16 years. As expected, this option produced the highest number of over-reporters at 3%.

**Table 1:** Comparison of prevalence of misreporters* based on three criteria options (*n* = 4826)*.*

	Option 1		Option 2		Option 3	
	Under-reporters	Over-reporters	Under-reporters	Over-reporters	Under-reporters	Over-reporters
Boys						
2–3 years	1.2%	3.1%	1.7%	1.3%	0.6%	0.9%
4–8 years	1.9%	2.9%	2.6%	1.8%	1.6%	0.8%
9–13 years	5.2%	2.9%	8.2%	1.5%	8.2%	1.4%
14–16 years	8.2%	3.0%	10.7%	2.1%	10.4%	2.0%
Girls						
2–3 years	1.1%	5.5%	1.3%	3.6%	1.1%	2.3%
4–8 years	1.3%	2.7%	2.4%	2.3%	1.3%	1.3%
9–13 years	6.1%	3.3%	9.5%	2.6%	9.1%	2.6%
14–16 years	15.3%	2.0%	15.3%	1.7%	14.2%	1.7%
Total	5.0%	3.0%	6.7%	2.1%	6.0%	1.6%

* Population weights applied; Option 1: excludes misreporters after application of cut-offs based on PAL of 1.55; Option 2: excludes misreporters after application of cut-offs based on individually measured PAL or estimated PAL (1.65); Option 3: excludes misreporters after application of cut-offs based on individually measured PAL or estimated PALs of 1.50 (lower limit) and 1.80 (upper limit).

When the cut-offs were based on individually measured PAL or estimated PAL of 1.65 (option 2), the prevalence of under-reporting was higher at 6.7%. Under-reporting among 2 to 8 year olds was less than 3%, which increased to 11–15% among 14–16 year olds. The overall rate of over-reporting was 2.1%.

The criteria based on individual PAL with wider estimates for those who had no measured PAL (option 3) produced the lowest number of over-reporters in all age groups and overall (1.6%). Compared to option 2, there were few differences in the levels of under- and over-reporting for boys and girls aged 9 to 16 years. For boys and girls aged 2 to 8 years (all with estimated PAL, not measured PAL) the proportion of under-reporting and over-reporting was lower using this option compared to option 2 which used narrower confidence limits.

Among the subset of children with measured PAL, a fourth option of direct comparison of EI to TEE was applied to identify misreporters. This option resulted in 3.3% of children being identified as under-reporters and 6.8% as over-reporters. The percentage of under-reporters using this option was lower than for the other 3 options (using Goldberg cut-offs) when examining the same subset of children (3.3% *versus* 7.8%–10.5%), while the percentage of over-reporters was higher (6.8% *versus* 1.7–2.7%). The overall mean ratio of EI to TEE was 0.97 (SD 0.37).

### 3.2. Impact of Misreporters on the Relationship between EI and BMI

To examine the impact of misreporting on the association between reported EI and BMI, several regression models were explored both including and excluding misreporters. Linear regression models which included misreporters found no significant associations between reported EIs and BMI *z*-score (Table 2). However, when misreporters were excluded (using any of the three options), highly significant associations were found between reported EIs and BMI *z*-score, when adjusted for age and gender. The model with the highest regression coefficient between reported EI and BMI *z*-score was based on excluding misreporters using option 2, *i.e.*, using individual PAL where available and mean PAL (1.65) for other children.

These models were also analysed using only children who had participated in the 4 day physical activity recall diaries, *i.e.*, those aged 9 years or older. The results of these models were very similar to the models that used measured and estimated PALs (data not shown). 

**Table 2:** Linear regression models showing variables associated with energy intake (MJ); including and excluding misreporters *.

	Include all		Option 1		Option 2		Option 3	
	B	*p*	B	*p*	B	*p*	B	*p*
Age (year)	0.332	<0.001	0.374	<0.001	0.381	<0.001	0.376	<0.001
Sex	−1.442	<0.001	−1.359	<0.001	−1.431	<0.001	−1.439	<0.001
BMI *z*-score	−0.034	0.41	0.152	<0.001	0.168	<0.001	0.157	<0.001
	R^2^*=* 0.25		R^2^*=* 0.39		R^2^*=* 0.38		R^2^*=* 0.36	
	*n =* 4792		*n =* 4408		*n =* 4373		*n =* 4426	

* Population weights applied; Option 1: excludes misreporters after application of cut-offs based on PAL of 1.55; Option 2: excludes misreporters after application of cut-offs based on individually measured PAL or estimated PAL (1.65); Option 3: excludes misreporters after application of cut-offs based on individually measured PAL or estimated PALs of 1.50 (lower limit) and 1.80 (upper limit).

### 3.3. Characteristics of Misreporters

Characteristics associated with misreporting in the 2007 Australian Children’s Survey are shown in Table 3. Misreporting was most common in the higher age groups especially in the 14–16 year age group with girls more likely to misreport than boys. There were also significant differences in the extent of misreporting by BMI (and BMI *z*-score) for boys and girls. Children who under-reported their EIs were more likely to have a higher BMI compared to plausible reporters. Conversely, children who over-reported EIs were more likely to have a lower BMI. Significant differences were also found between misreporting and PAL, with under-reporters having higher PALs and over-reporters having lower PALs. Unfortunately measured PALs were only available for children aged 9 years and over. 

Parental education was also significantly related to misreporting; under-reporting was higher among children whose primary caregiver had lower levels of education. A slight but significant difference was found between misreporting and area of residence, with children living in urban areas being more likely to under-report their EI. In addition, children who reported not feeling well on the day of the survey were more likely to be under-reporters than children who did not report this. Overall, 3.0% of children in the survey reported not feeling well on the day of the survey.

No significant differences in misreporting were found between weekdays and weekend day. 

**Table 3:** Characteristics of misreporters* (identified based on option 2).

Characteristic	Under-reporters	Plausible reporters	Over-reporters	*p*
Total, % (*n*)	6.7%	91.3%	2.1%	
Energy intake (MJ), mean (SE)	5.03 (0.08)	8.50 (0.04)	16.11 (0.50)	<0.001
Age group, %				
2–3 years	1.5%	96.0%	2.5%	<0.001
4–8 years	2.5%	95.4%	2.1%	
9–13 years	8.8%	89.2%	2.0%	
14–16 years	13.0%	85.1%	1.9%	
Gender, %				
Boys	6.1%	92.2%	1.7%	0.043
Girls	7.3%	90.3%	2.4%	
Parental education, %				
School/certificate	7.2%	90.2%	2.5%	0.010
Diploma/degree	6.0%	92.5%	1.5%	
Area of residence, %				
Urban	7.3%	90.7%	2.0%	0.034
Rural	5.3%	92.5%	2.2%	
Day of the week, %				
Weekday	6.6%	91.8%	1.6%	0.133
Weekend day	6.7%	90.7%	2.6%	
BMI, mean (SE)				
Boys	22.6 (0.46)	18.3 (0.07)	18.2 (0.37)	<0.001
Girls	22.7 (0.41)	18.6 (0.08)	17.3 (0.42)	<0.001
PAL ^, mean (SE)				
Boys	1.81 (0.03)	1.71 (0.01)	1.63 (0.06)	<0.001
Girls	1.67 (0.02)	1.60 (0.01)	1.56 (0.03)	0.001
Unusual intake on survey day, %				
Feeling unwell	29.5%	69.2%	1.4%	<0.001
No comment on wellness	6.0%	92.0%	2.1%	

* Population weights applied; ^ For measured PAL only, children 9 years and over.

## 4. Discussion

This study explored some of the issues related to misreporting of EI in the 2007 Australian Children’s Survey; including identifying potential misreporters, describing the characteristics of misreporters, and examining the impact of including or excluding misreporters on the relationship between EI and BMI. The 2007 Australian Children’s Survey is a representative national survey of 4800 Australian children based on age, gender and region. Strengths of the study included the availability of measured heights and weights, as well as validated PAL data for children aged 9 years and over. 

### 4.1. Identifying Misreporters

Misreporters were identified based on a variety of criteria. Options 1 to 3 used the Goldberg criteria with different cut-offs depending on the PAL value used. Option 1 assumed an activity level of 1.55 for all children based on the value defined by FAO/WHO/UNU as that which represents a sedentary level of energy expenditure [11]. This PAL was traditionally used in the Goldberg equations to identify potential under-reporting if no individual PAL was available [9] but at the expense of sensitivity [12]. The Main Findings Report of the 2007 Australian Children’s Survey [20] used a single PAL of 1.55 to estimate under-reporting and found the same estimates as in option 1 of our study, with less than 2% of the younger children (2–8 years); 5–6% of children aged between 9–13 years and 8–16% of the older children (14–16 years) having potentially implausibly low intakes. Although under-reporters were identified in this survey report, they were not excluded in further analysis. The application of such a conservative PAL (1.55) to this population of normally active children, however, is likely to result in an underestimation of the prevalence of under-reporting. For example, using a PAL of 1.55 for a child with high energy expenditure will lead to a very conservative lower cut-off value and may therefore fail to identify under-reporting. Conversely, the prevalence of potential over-reporting is likely to be overestimated as the upper cut-off at a PAL of 1.55 will be too low for those with high energy expenditure. As expected, when compared to the other options, applying a PAL of 1.55 for all children resulted in a lower proportion of under-reporters and a higher proportion of over-reporters being identified. 

Option 2 applied individually measured PAL if available, and applied estimated activity levels of 1.65 (based on group mean) for children who had not participated in the physical activity diary data collection. The use of individual PAL is preferable to using a single PAL as it increases the sensitivity of the Goldberg equations [12]. Option 2 identified the highest proportion of misreporters (8.8%), and in particular under-reporters, compared to the other options (8.0% and 7.6% for options 1 and 3, respectively) although the differences were relatively minor.

Option 3 was similar to option 2 in that individual PAL were applied to the Goldberg equations where available but the estimated PALs were based on wider cut-offs (using PAL of 1.50 for lower limit and 1.80 for upper limit) for children with no measured PAL. The use of wider cut-offs results in a larger proportion of the sample being considered plausible reporters. A similar method was used in the “What America Drinks” report where the lowest cut-off was based on a sedentary level of physical activity and the upper cut-off was based on a highly active level of physical activity [21]. Using this approach, the range of predicted energy requirements for each person is much greater than a range associated with only one activity level, and consequently classifies more respondents as plausible reporters. One of the drawbacks of this method is that it is less effective in correcting the distortion in the biological relationship between EI and weight status, and therefore weakens the relationship between EI and BMI [7].

In a similar comparative study undertaken in adults in the United Kingdom, Rennie *et al.* [2] found that using individualised estimates of energy requirements were preferable to those using a single Goldberg cut-off in evaluating under-reporting in the 2000 National Diet and Nutrition Survey. 

Option 4, direct comparison of EI to TEE (using physical activity diaries as a measure of PAL) may be a better option for identifying misreporters as the CV for TEE is assessed in absolute terms using the survey data, instead of published CV values. Nevertheless, the variation in EI, BMR and PAL were very similar to the values used in the Goldberg equations leading to comparable results. This option resulted in fewer under-reporters but slightly higher numbers of over-reporters. At the group level, the mean EI to TEE ratio was 0.97 suggesting that reporting on average was adequate for those who had a measured level of PAL [22].

The level of UR in this study was relatively low compared to that of other surveys that compared EI to EE using the DLW method [22]. This is most likely due to the wide CL that were applied to detect misreporters as variability was relatively high for energy expenditure (CV 17.8%) and EI (CV 23%) and only one day of dietary intake was assessed. 

### 4.2. Best Model to Assess Relationships between Reported EI and BMI

Our assessment of the impact of misreporting on the relationship between EI and BMI shows that the exclusion of misreporters, as identified using any of the 3 options described, resulted in the emergence of a significant positive relationship between EI and BMI, adjusted for age and gender. Ideally, a tight definition of misreporters should be applied to examine this relationship. Option 2 (application of cut-offs to individual PAL) may be the preferred method with the regression model showing the highest regression coefficient for BMI *z*-score of any of the 4 models. The overall model (with age, gender and BMI *z*-score) explained approximately 40% of the variability of the data compared to only 25% of variability if misreporters were included in the model. These results highlight the need to consider the impact of misreporters on the validity of the data. 

Nevertheless, examining relationships between EI and BMI in cross-sectional datasets has severe limitations. BMI is a key physiological predictor of total energy expenditure and thus children with a higher weight status are likely to have a higher EI if they are in energy balance. Although a biological relationship between EI and weight status is evident in the long term, such associations may not be as apparent in cross-sectional studies. In addition, the use of a single 24-h recall is not a measure of usual dietary intake among individuals. Associations between dietary intake and weight status are best assessed in longitudinal datasets.

Our findings are consistent with those of several other studies undertaken in adults which reported that inaccurate reports of EI obscured relationships between diet and health [5,7,8,23]. Huang *et al.* [8] found that implausible EIs impacted relationships between BMI and dietary factors such as EI, meal portion size, energy consumed per meal and eating frequency. A recent large Canadian study similarly found that excluding under- and over-reporters led to stronger relationships between BMI and EI and this was confirmed for specific age and gender subgroups [7].

### 4.3. Characteristics of Misreporters

Misreporting was associated with several sociodemographic characteristics including age, gender, parental education, area of residence, BMI, PAL and feeling unwell on the survey day. Older children, especially adolescent girls were the highest under-reporters. These findings are consistent with those of Livingstone and Black [22] in a comprehensive review of characteristics associated with under-reporting. As reporting is the responsibility of the parent or caregiver for the younger children, there is a lower likelihood of under-reporting. Older children and adolescents (9–16 years) self-reported their intake and levels of under-reporting started to rise. For adolescents, under-reporting was highest which could be due to a number of factors including increased energy requirements leading to a greater amount of food to recall, unstructured eating, concerns with self image and rebellion against authority. As in adults, children with a higher BMI were more likely to under-report their EI which may indicate poor self-monitoring of food intake or denial in this group. Conversely, children with a lower BMI were more likely to over-report their EI.

Misreporting was also associated with PAL; children with higher PAL tended to under-report while those with a lower PAL tended to over-report. Under-reporting of intakes among children with a higher PAL could be due to children having higher energy requirements and not accurately reporting the frequency of consumption or the portion sizes of large amounts of foods. 

Urban children and children whose primary parent had a lower education level were also more likely to be under-reporters. Inconsistent results are found in the literature, with higher levels of under-reporting found among those with poor literacy skills in the less well educated, as well as among the more diet conscious people with better education [22]. 

Feeling unwell on the day of the survey was strongly associated with misreporting; 30% of children who felt unwell under-reported their EI compared to 6% of children who did not report feeling unwell. This is likely to be a result of under-eating rather than under-recording. However to determine the degree of under-eating, body weight must be monitored, usually in longer term studies. Consistent with our findings, Rennie *et al.* [2] found that the exclusion of subjects who reported their eating being affected by diet or illness during the recording period resulted in lower levels of under-reporting. 

### 4.4. Limitations

There are a number of limitations to be noted especially in regards to dietary recalls which are prone to subject selection bias, errors in portion size estimation and recording bias. Translating food intake data into highly accurate EI is also problematic due to the inherent limitations associated with food composition databases. Although AUSNUT 2007 was developed specifically for this study, the nutrient data were derived from a range of sources, and the nutrient composition of foods can vary substantially between batches and brands [15]. Additional problems relating to measuring children’s diets include issues of literacy, limited food recognition skills, memory constraints and concentration span. Dietary data were collected from the primary care-giver for children aged 2–8 years whereas children aged 9 years and older reported their own dietary intake. Biased reporting may have occurred by both care-givers and children although an assessment of the types of food that were misreported is beyond the scope of this study. In addition, there are a number of assumptions and limitations pertaining to the Goldberg cut-offs. The Goldberg equations assume that body weight is stable, which may not be the case for growing children although the extra amount of energy required for growth in children after age two is small at approximately 1% of energy expenditure [24] and would have little impact on the results. The day-to-day variation in EI was assumed to be relatively high (CV 23%) and based on one day of dietary recall, this translated into wide CL. Only extreme degrees of misreporting can be identified using this method. Lastly, children who did not participate in the MARCA were assigned a PAL of 1.65 which was the average PAL of the children who did participate in the MARCA. Using this ‘estimated’ value was therefore not a true indication of their PAL and may have resulted in some misclassification of energy reporting.

## 5. Conclusions

Misreporting of EI is present among various subgroups of the 2007 Australian Children’s Survey. Under-reporting was more evident in older children, especially adolescent girls; children with higher BMI; children with higher PAL; urban children; children whose primary parent had a lower education level; and in children who felt unwell on the day of the survey. Over-reporting was more common among children with a lower BMI and those with a lower PAL. Misreporting influenced the relationship between reported EI and BMI and users of this dataset should consider excluding misreporters when evaluating potential diet-BMI associations in future analysis.
